# Transcriptional analysis of porcine circovirus-like virus P1

**DOI:** 10.1186/s12917-014-0287-3

**Published:** 2014-12-02

**Authors:** Li-bin Wen, Feng-zhi Wang, Kong-wang He, Bin Li, Xiao-min Wang, Rong-li Guo, Jian-ping Xie

**Affiliations:** Jiangsu Academy of Agricultural Sciences · Key Laboratory of Veterinary Biological Engineering and Technology, Ministry of Agriculture · National Center for Engineering Research of Veterinary Bio-products, Institute of Veterinary Medicine, Nanjing, 210014 China

**Keywords:** Porcine circovirus like virus P1, Transcriptional analysis, Northern blotting, RACE

## Abstract

**Background:**

Recently identified porcine circovirus-like virus P1 has the smallest DNA viral genome. In this study, we identified the viral genes and their corresponding mRNA transcripts.

**Results:**

The RNAs of P1, synthesized in porcine kidney cells, were examined with northern blotting and PCR analyses.

Eight virus-specific RNAs were detected. Four mRNAs (open reading frames (ORFs) 1, 2, 4, and 5) are encoded by the viral (−) strand and four (ORFs 3, 6, 7, and 8) are encoded by the viral (+) strand. All proteins encoded by the ORFs of the P1 virus are less than 50 amino acids in length, except that encoded by ORF1 (113 amino acids).

**Conclusions:**

We show a very complex viral transcription pattern in P1-infected cells.

## Background

Porcine circovirus-like virus P1, the smallest DNA virus in terms of its genome size, was isolated from pigs with postweaning multisystemic wasting syndrome (PMWS) [1,2]. Its genome consists of a single-stranded, covalently closed circular molecule of only 648 nucleotides. Electron micrographs showed that the P1 virion is a non-enveloped particle with a diameter of about 25 nm.

PMWS, a swine disease that occurs worldwide, was first identified in a swine herd in Canada [3,4]. It is characterized by progressive weight loss, respiratory symptoms, and jaundice, and has had a significant economic impact on the pig industry [5]. Porcine circovirus type 2 (PCV2) is considered the causative agent of PMWS. PCV2, a member of the genus *Circovirus* in the family *Circoviridae*, is a nonenveloped, icosahedral virus with a single-stranded circular DNA genome of approximately 1.7 kb [6].

The overall DNA sequence homology between the P1 isolates is greater than 99%, whereas the sequence homology between P1 and PCV2 isolates is 32.6%—35.5%, although P1 has high nucleotide sequence homology compared with PCV2 isolates. A phylogenetic analysis suggested that P1 isolates are closely related to PCV2 isolates.

Epidemiological studies have reported that 19% of swine in China are P1-viremic [7]. Data from pigs transfected with an infectious DNA clone of P1 indicated that P1 can also cause PMWS-like clinical disease in pigs [2]. The genomic sequences of a number of P1 isolates have been determined. P1 consists of three potential open reading frames (ORFs) larger than 75 nucleotides (nt), analyzed with the DNAMAN software. The genome of P1 is ambisense [8], and ORFs 1 and 2, are encoded by the viral (−) strand, whereas ORF3 is encoded by the viral (+) stranded. The genetic basis for P1 pathogenicity cannot be determined from the genomic sequences, although the genomic sequences of many P1 viral isolates have been determined. So far only a few transcriptional analyses of PCV2 have been reported, but up to 13 RNAs have been detected [9-14]. Nine of these RNAs (CR, Rep, Rep’, Rep3a, Rep3b, Rep3c, NS515, NS672, and NS0) of PCV2 have also been identified in these studies [11]. Until now, no transcriptional analysis of P1 has been reported. To better understand the mechanism of P1 pathogenesis, it is essential to know how many genes it expresses. In this study, we detected and mapped eight virus-specific RNAs in P1-transfected PK15 cells.

## Methods

### P1 virus genomic DNA

The full-length genome of the P1 virus JSNJ was isolated from the serum of a piglet with PMWS. All sample collection was conducted between December 2013 and January 2014 in Jiangsu Province, China. Permission to collect the study samples was granted by the pig farms. All procedures involving animals throughout the study were approved by the Committee on the Ethics of Animal Experiments at the Institute of Veterinary Medicine, Jiangsu Academy of Agricultural Sciences, China. The viral sequences were determined after the genome was amplified with PCR. The genome was digested singly *Bam*HI, tandem dimers were ligated with T4 DNA ligase, and the dimeric genomes inserted into the *Bam*HI site of the pBluescript SK (pSK) vector (Stratagene). The cloned constructs were used for *in vitro* transfection assays.

### Cell culture and transfection

A PK15 cell line [2] free of PCV2, PCV1, and mycoplasma contamination was maintained in RPMI 1640 medium supplemented with 10% fetal calf serum and 0.01% penicillin–streptomycin in 5% CO_2_ at 37°C. The cells were transfected with Lipofectamine^TM^ 2000 (Invitrogen), according to theprotocol recommended by the manufacturer. DNA (4 μg) was used to transfect 10^6^ cells in six-well plates. The transfected cells and the culture media were harvested at different times (0, 12, 24, 48, 72, 96, and 120 h) and frozen at −80°C before RNA extraction. The pSK vector control was used to check for any nonspecific responses, and no specific bands were detected with either northern blotting or reverse transcription polymerase chain reaction (RT-PCR).

### RNA isolation and preparation

Total RNA was extracted from the transfected cells with TRIzol Reagent (invitrogen), according to the manufacture’s instructions. The quality of the RNA template was assessed by the ratio of 28S:18S RNAs on a denaturing formaldehyde agarose gel after it was stained with ethidium bromide. The RNA concentrations were determined spectrophotometrically at 260 nm.

The total RNAs isolated at selected times for northern blotting analysis were not treated with DNaseI, and the vector DNA was used as an internal control. Any residual DNA was removed from the RNA samples for both RT-PCR and the random amplification of cDNA ends (RACE) with the TURBO DNA-free^TM^ Kit (Ambion), according to the manufacture’s instructions. A control PCR reaction without the RT step was also conducted to ensure that the input P1 plasmid DNA was completely digested by DNaseI.

For RT-PCR, 1 μg of RNA was reverse transcribed at 42°C for 1 h using avian myeloblastosis virus (AMV) reverse transcriptase and an oligo(dT)_18_ primer, according to the protocol of the manufacturer (TaKaRa, China).

5′- and 3′- RACE products were generated from first-strand cDNA synthesized from 1 μg of total RNA in a 10 μL reaction mixture using the SMARTer^TM^ RACE cDNA amplification kit (Clontech catalog no. 634923), according to the manufacturer’s protocol. In this reaction, RNA was reverse transcribed with SMARTScribe Reverse Transcriptase at 42°C for 90 min. The 3′-CDS primer A or 5′-CDS primer A and SMARTer IIA oligonucleotide were used to synthesize 3′-RACE-Ready cDNA and 5′-RACE-Ready cDNA, respectively.

### Northern blotting analysis

Single-stranded RNAs were prepared by *in vitro* transcription using T7 RNA polymerase. The P1 molecular DNA clone was used as the template for PCR to generate the (+) and (−) P1 transcripts. The primer sets were probe R (F1: 5′-GCGCTAATACGACTCACTATAGGGATCTTCAACACCCGCCTCT-3′, R1: 5′-GGATATTGTAGTCCTGGTCGTAT-3′), and probeF (F2: 5′-ATCTTCAACACCCGCCTCT-3′, R2: 5′-GCGCTAATACGACTCACTATAGGGGGATATTGTAGTCCTGGTCGTAT-3′). Two digoxigenin (DIG)-labelled viroid-specific riboprobes were synthesized using the DIG RNA Labelling Kit (SP6/T7) (Roche Applied Science), as recommended by the manufacturer.

The RNAs were separated with 1% formaldehyde agarose gel electrophoresis (25 V overnight) and electroblotted (400 mA for 1 h) onto positively charged nylon membranes (HyBond N+, Amersham Pharmacia Biotech) by capillary transfer in 20 × saline sodium citrate (SSC) and immobilized for 2 h at 80°C. The membranes were prehybridized for 2 h at 68°C with Roche DIG Easy Hyb and then hybridized overnight at 68°C in DIG Easy Hyb containing the denatured probe. After hybridization, the membranes were washed twice (15 min each) in 0.1 × SSC/0.1% sodium dodecyl sulfate (SDS) solution at 68°C, equilibrated for 2–5 min in washing buffer, blocked in blocking solution with gentle agitation for 1 h, incubated for 30 min in alkaline-phosphatase-conjugated anti-DIG antibody, washed twice (15 min each) in washing buffer, and incubated for 5 min in CSPD® Substrate (Roche Applied Science). Following the chemiluminescent detection procedure, the membranes were exposed to X-ray films for 10–30 min.

An RNA molecular weight marker I, DIG-labeled (0.3–6.9 kb) (Roche Applied Science) was used as the size standard.

### RT-PCR

PCR was used to amplify the cDNA generated from about 20 ng of total RNA in triplicate, in a final volume of 25 μL, using specific primers. An aliquot (10 μL) of the reaction mixture was analyzed with gel electrophoresis after 35 cycles of amplification. The primers used for RT-PCR were 170F: 5′-TTTGTTATTTGGTTGGAAGTAATCAATAGT-3′; 462R: 5′-CCAGGAGGGGGGACCAACAAA-3′; 485F: 5′-AATCTCATCATGTCCACCGCCCAGGAG-3′; 579R: 5′-GGCATCTTCAACACCCGCCTC-3′; 288F: 5′-GGTCATAGGTTTGGGCCGTGG-3′; 577F: 5′-GCCATTTTTCCTTCTCCAACG-3′; and 36R: 5′-TTAATCTTAAGGGCCCCCCAC-3′.

### RACE

5′- and 3′-RACE PCR amplification were performed using the 5′-RACE-Ready and 3′-RACE-Ready cDNAs, respectively. RACE-PCR was performed with the Universal Primer A mix (UPM) from the SMARTerRACE cDNA Amplification Kit, and the primers appropriate to 5′-RACE or 3′-RACE. The conditions for amplification were those recommended by the manufacturer: five cycles of denaturation at 94°C for 30 s, annealing-extension at 72°C for 3 min; followed by five cycles at 94°C for 30 s, 70°C for 30 s, and 72°C for 3 min; and finally, 25 cycles of 94°C for 30 s, 68°C for 30 s, and 72°C for 3 min. All the products were analyzed with gel electrophoresis on 2% agarose gels, purified after gel elution, cloned into the pMD-18 T vector (TaKaRa, China), and sequenced. Two controls were also used to exclude the possibility of nonspecific amplification. One control reaction was performed with only one viral-specific primer, and the other was performed with UPM alone. No bands were amplified in either control.

The primers used for RT-PCR were 267F: 5′-CGGGAGGAGTAGTTACATATGGG-3′; 540F: 5′-TATATCCGAAGGTACGGGAGAGG-3′; 536R: 5′-TCAAGGCTACCACAGTCAGAACG-3′; 640F: 5′-AGTGGATCCTCATTTAGGGTTTAAGTGG-3′; 299R: 5′-AAACCTATGACCCATATGTAACT-3′; 343R: 5′-CCCTGTTATTCTAGATGATAACTTTG-3′; 425R: 5′-CCCACTACAGAATAAGAAAGGTTAAG-3′;248R: 5′-CTTCTCCTACCACTCCCGTTACT-3′; 562R: 5′-CCTCTCCCGTACCTTCGGATATA-3′;541F: 5′-ATATCCGAAGGTACGGGAGAGGC-3′; 30R: 5′-CTAAAGACCCCCCACTTAAACCC-3′; and 285F: 5′-ATGGGTCATAGGTTTGGGCCGTG-3′.

### Virus and expression of recombinant ORF1 protein

The purified P1 virus was produced as previously described [15]. Briefly, PK15 cells were transfected with the P1 DNA clone and harvested at 96 h. The cells were frozen and thawed three times, and centrifuged in two rounds of CsCl density gradient ultracentrifugation. The P1 virus was collected by aspiration and dialysis.

To construct an expression plasmid encoding the ORF1 protein, ORF1 of P1 was amplified by PCR using primers 5′-GGC*GGATCC*ATGAGATTTAATATTGACGAC-3′ and 5′-ATA*CTCGAG*GCCAAAGCTGATTCCTTTTG-3′ (the italics sequences identify the *Bam*HI and *Xho*I sites). The PCR products were cloned into the pMD-18 T vector, doubly digested with *Bam*HI and *Xho*I, and cloned into the prokaryotic expression vector pET-32a (+) (Novagen, Germany). The resulting recombinant expression plasmid was used to transform *Escherichia coli* DH5α cells and was identified by restriction enzyme digestion and DNA sequencing.

To express the cloned ORF1, *E. coli* BL21 (DE3) pLys cells were transformed with the pET32a–ORF1 plasmid. Single colonies of transformants were grown in Luria–Bertani medium at 37°C to an optical density at 600 nm (OD_600_) of about 0.4–0.6, and isopropylthio-β-d-galactopyranoside was added to a final concentration of 1 mM. After induction at 20°C for 12 h, the bacteria were collected by centrifugation at 5000 × g for 10 min and lysed by boiling for 10 min. The fusion proteins from the whole-cell lysates and the virus purified by CsCl gradient centrifugation were analyzed with SDS polyacrylamide gel electrophoresis (PAGE) and immunoblotting, respectively. The rabbit anti-P1 hyperimmune serum used for immunoblotting was prepared as described previously [2].

## Results

### Molecular cloning of the P1 genome

**Figure 1:**
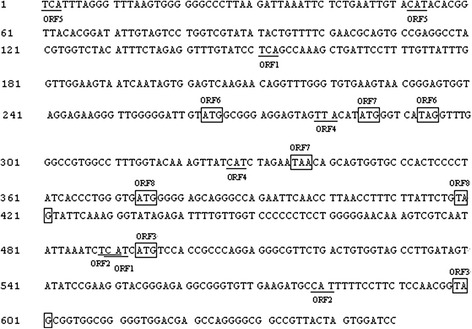
**Nucleotide sequence of the 648-nt P1/JSNJ circular genome [GenBank:KJ612072].** The initiation codons and termination codons of the ORFs transcribed to the right are shown in boxes, whereas those of the ORFs transcribed to the left are marked with underlining.

The complete genomic DNA sequence of the P1 virus was isolated from the serum collected from a pig in China suffering from PMWS using PCR and primer sets F and R, which each contain a *Bam*HI site [7]. A P1 clone, designated P1/JSNJ, was selected and sequenced. The genomic sequence of P1/JSNJ was 648 nt long [GenBank:KJ612072] and is presented in Figure 1. This sequence is more than 99% homologous to other P1 sequences in the GenBank database. After restriction enzyme digestion and ligation with T4 DNA ligase, the tandem dimers were inserted into the *Bam*HI site of the pSK vector.

### Northern blotting analysis

**Figure 2:**
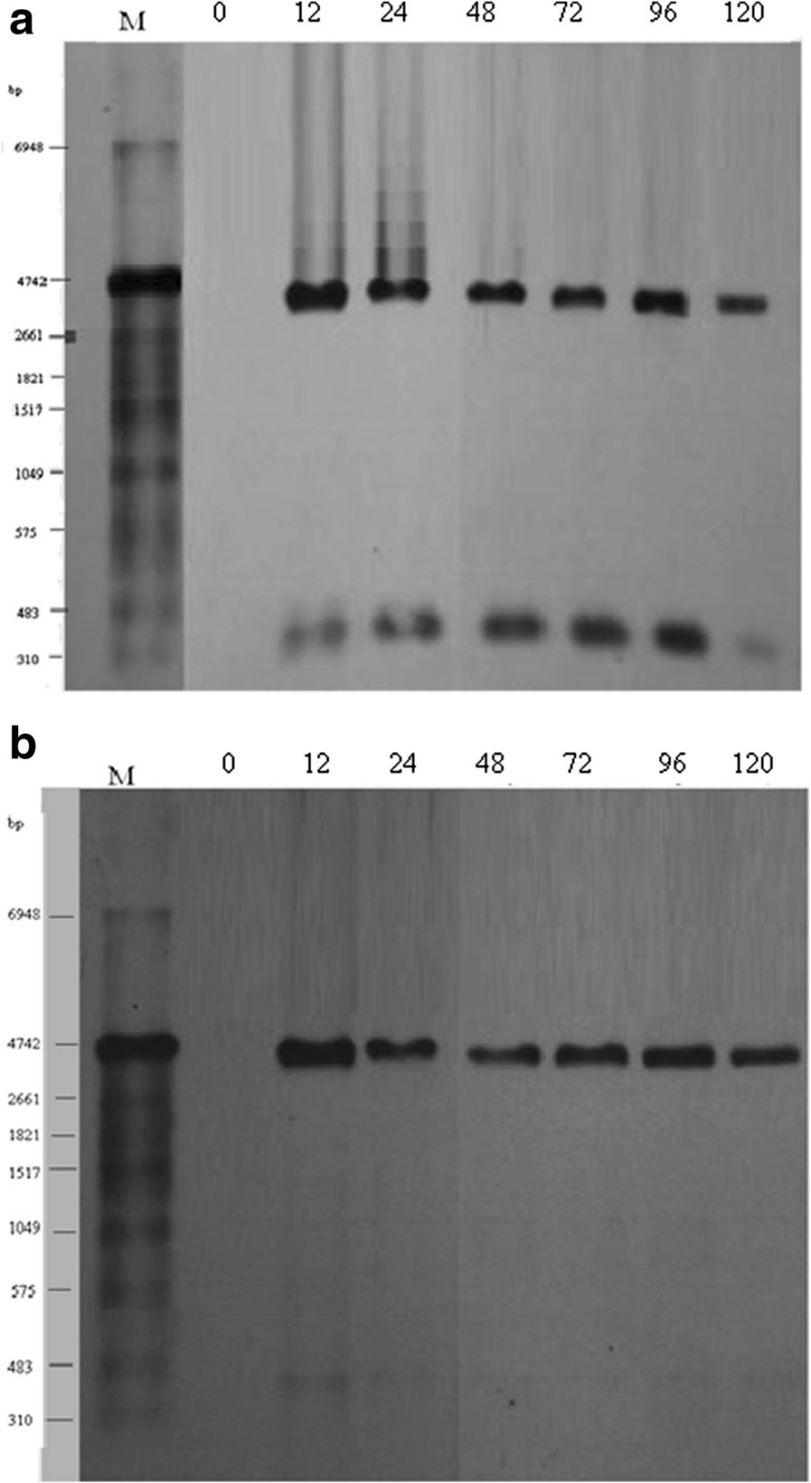
**Northern blotting analysis of RNAs from PK15 cells transfected with a molecular clone of the P1 virus with two copies of the genome.** Total cellular RNAs extracted at different times after transfection are listed at the top of each lane. **(a)** The riboprobe hybridized to the RNAs transcribed in the leftward direction. **(b)** The riboprobe containing the sequence complementary to the RNAs transcribed in the rightward direction.

This experiment was performed with two single-stranded riboprobes to determine the orientation and the relative abundance of the viral RNAs. Total cellular RNAs isolated at the designated times after transfection were hybridized with the two riboprobes. Probe F containing the DNA sequence of nt 67–576 hybridized to the 380 nt RNA corresponding to ORF1 and the internal control fragment, indicating that ORF1 is transcribed in the left-ward orientation. The 380-nt RNA was observed from 12 h after transfection and increased in a time-dependent way. The 96 h sample gave the strongest signal (Figure 2a). Probe R containing the DNA sequence at nt 576–67 (complementary to RNAs transcribed in the rightward orientation) hybridized to the 350-nt RNA (ORF3) and the internal control band. The 12 h sample gave the strongest signal, but there was no obvious relationship between the amount of ORF3 RNA and time (Figure 2b).

### PCR analysis

At least two virus-specific RNAs were synthesized in PK15 cells transfected with a dimers of the P1 virus genome, as demonstrated with northern blotting. To explore the minor RNAs synthesized from P1, the RNA samples were assayed with both RT-PCR and RACE. The PCR products were analyzed with gel electrophoresis under standard low-voltage conditions, and after the resulting DNA fragments were separated, they were cloned into the pMD-18 T vector. At least six clones of each PCR product were selected for sequencing.

### RT-PCR analysis

Apart from ORF1 RNA and ORF3 RNA, another minor RNA (ORF5) was isolated.

**Figure 3:**
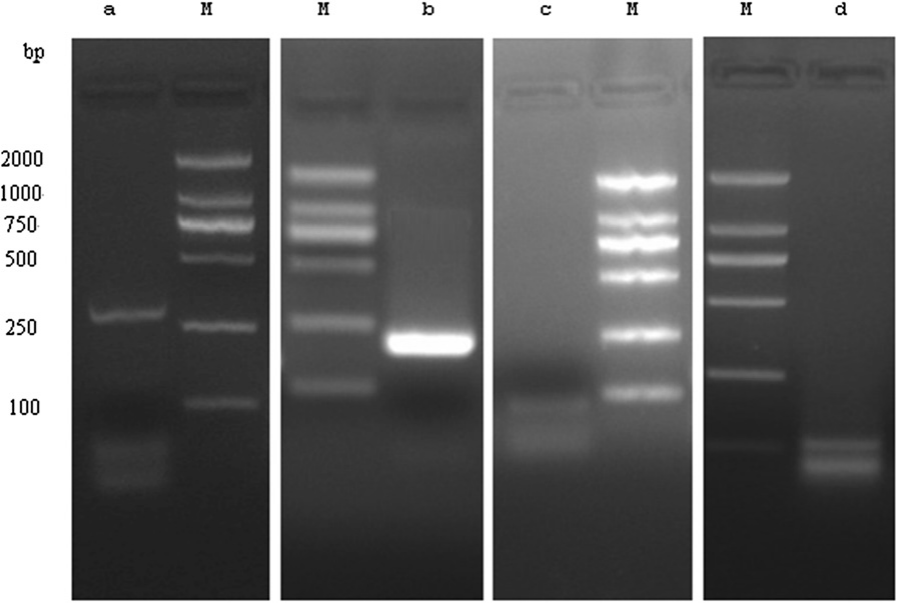
**RT-PCR was performed with DNaseI -treated RNA samples from 48 h after transfection. (a)** ORF1/ORF4; **(b)** ORF1/ORF4/ORF6/ORF7/ORF8; **(c)** ORF2/ORF3; **(d)** ORF5. M: DL2000 DNA marker.

ORF1 and ORF4. Primers 170F and 462R amplified a 300-bp band and the DNA sequence was collinear with the viral genome (Figure 3a).

ORF1, ORF4, ORF6, ORF7, and ORF8. Primers 288F and 462R amplified a PCR product of 180-bp and the DNA sequence was consistent with the viral genome (Figure 3b).

ORF2 and ORF3. Primers 485F and 579R amplified a single fragment of 100-bp and the DNA sequence corresponded to the genomic sequence (Figure 3c).

ORF5. PCR amplification using primers 577F and 36R produced a 100-bp DNA fragment and the DNA sequence was consistent with the viral genome (Figure 3d).

### RACE analysis

The 5′- and 3′- RACE products were generated from a first-strand cDNA template subjected to PCR amplification with 10× UPM and the designed primers.

### 5′ initiation sites

ORF1 and ORF4. PCR with UPM and 267F generated two clones with a 5′-initiation site at nt 457 and four clones with a 5′-initiation site at nt 506. We assumed that the former was used by ORF4 and the latter by ORF1.

ORF2. The 5′-initiation site at nt 648 was detected in a band of about 150 bp after PCR with the primer pair UMP and 540F.

ORF3. The 5′-initiation site (nt 364) for ORF3 was detected in a fragment of about 210 bp with PCR using the primer pair UMP and 536R.

ORF5. PCR with UPM and 640F generated three clones with the 5′-terminal nt at 82.

**Figure 4:**
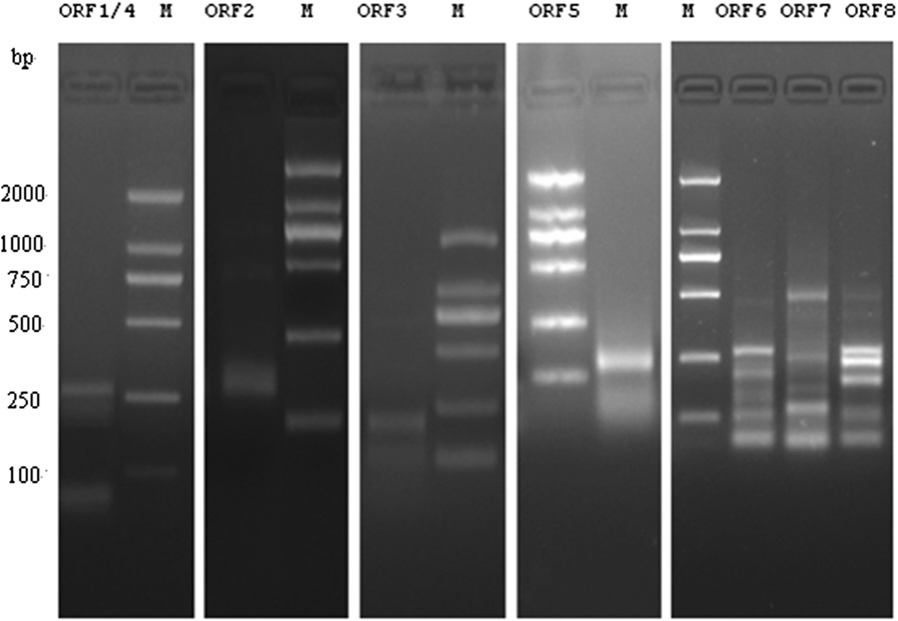
**RACE was performed with the selected primers and UPM to identify the 5′ ends of the transcripts.** M: DL2000 DNA marker.

ORF6, ORF7 and ORF8. PCR with UPM/299R, UPM/343R, and UPM/425R produced many bands. Sequence analysis of the 110-bp, 130-bp, and 200-bp fragments showed that four clones had a 5′-initiation nt at 232, three clones at nt 253, and five clones at nt 277, respectively. We assumed these sites were used by ORF6, ORF7, and ORF8, respectively (Figure 4).

### 3′ poly(A) sites

ORF1 and ORF4. PCR with UPM and 248R generated a 160-bp fragment that produced four clones with 3′-terminal at nt 125. Presumably, this poly(A) site is used by ORF1 and ORF4 RNAs.

ORF2. The 3′ poly(A) site at nt 481 was identified from a band of about 120-bp after PCR with UPM and 562R.

ORF3. The 3′ poly(A) site at nt 70 was identified from a fragment of about 220-bp after PCR with UPM and 541 F.

ORF5. The 3′ poly(A) site at nt 580 was identified from the 140-bp band after PCR with UPM and 30R.

**Figure 5:**
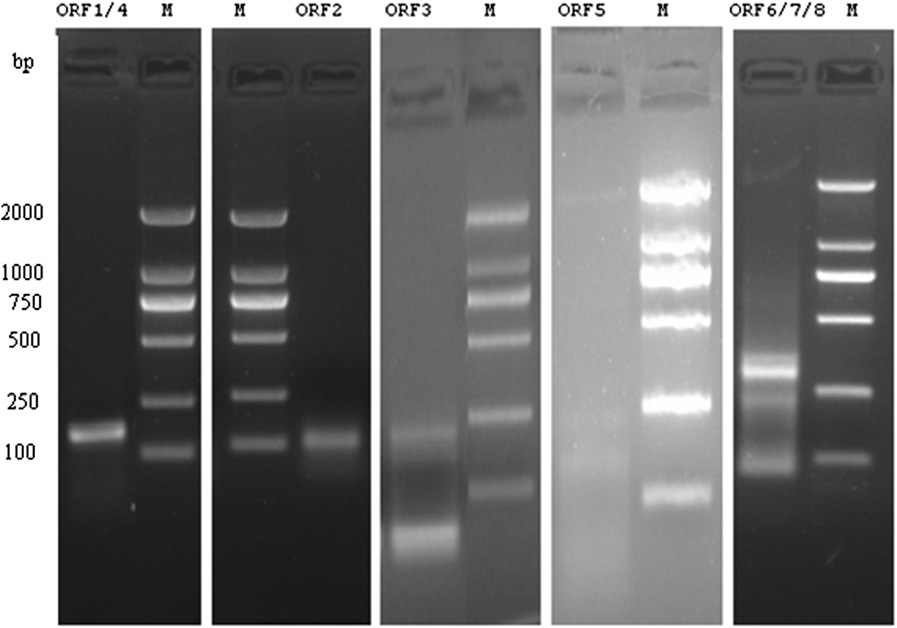
**Determination of the 3′ termini of the transcripts with RACE.** M: DL2000 DNA marker.

ORF6, ORF7 and ORF8. PCR with UPM and 285F generated a 210-bp fragment that produced four clones with 3′-terminal nt at 458. We inferred that this poly(A) site is used by ORF6, ORF7, and ORF8 RNAs (Figure 5).

**Figure 6:**
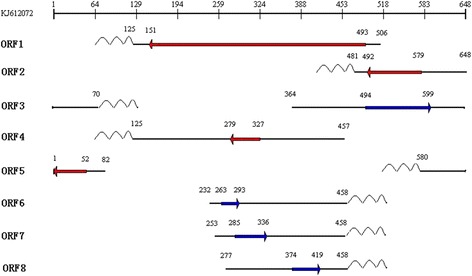
**Outline of the P1 RNAs.** A schematic representation of the P1 genome is presented at the top. Schematic representations of the P1 RNAs are shown. ~ ~ ~ indicates polyA. The numbers at both ends of each transcript indicate the first and last nucleotides of each transcript. The coding sequence of each transcript is shown in bold, bright colour, and their nt coordinates are indicated in the middle of the transcripts. The 5′ → 3′ direction is indicated by the arrow.

Each transcript is shown schematically in Figure 6.

### Major structural protein

**Figure 7:**
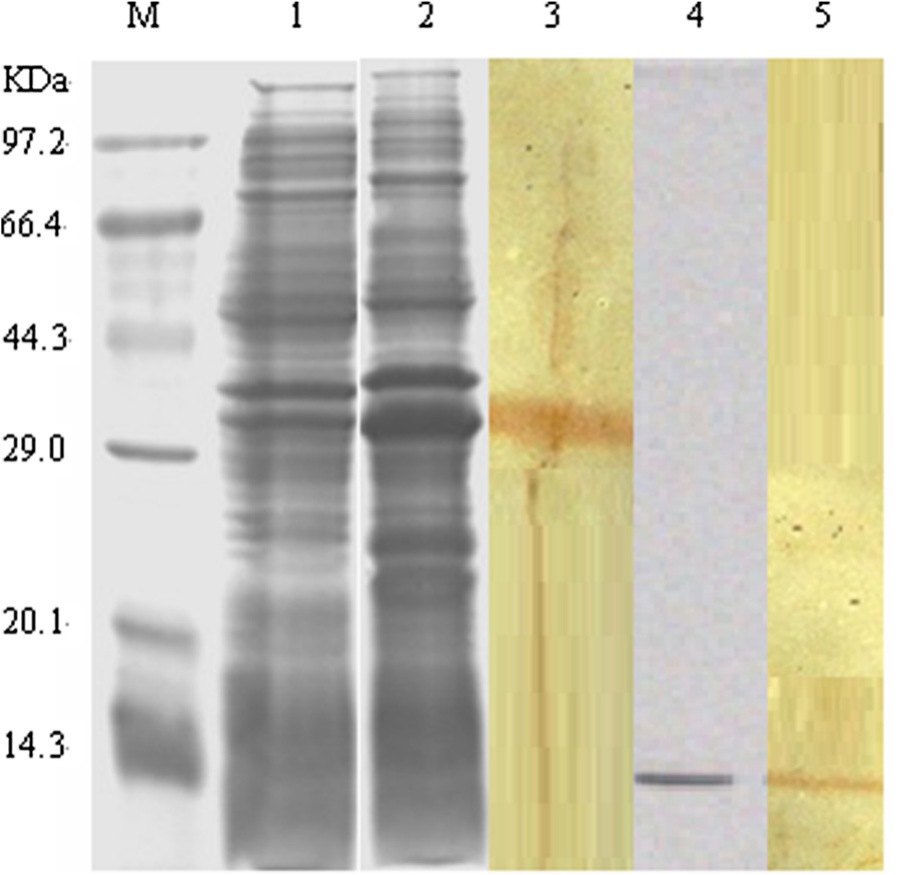
**Expression of the recombinant protein and the purified P1 particles were analyzed with SDS-PAGE and immunoblotting.** M: standard molecular marker; Lane 1: crude protein from noninduced BL21 cell lysate; Lane 2: crude protein from induced BL21 cell lysate; Lane 3: the fusion proteins reacting with polyclonal rabbit anti-P1 antibodies; Lane 4: gradient-purified P1 virus; Lane 5: the purified P1 virus after incubation with anti-P1 rabbit hyperimmune serum.

A single protein of approximately 12.5 kDa was detected in the CsCl gradient-purified P1 particles. The approximate molecular mass of the pET32–ORF1 fusion protein was 31 kDa, which corresponds to the theoretical molecular mass of the ORF1 protein (12.5 kDa). Both P1 particles and the fusion protein reacted strongly with a polyclonal rabbit anti-P1 antiserum on immunoblotting (Figure 7).

## Discussion

Whether the P1 virus is really a virus remains highly debatable in the circovirus research community. Some in the field speculate that P1 may represent a replication-competent subviral particle of PCV2, similar to another virus with a circular genome, TTV [16]. However, unlike the intragenomic rearrangement of PCV2 reported in our previous study [17-19], the P1 virus contains exogenous DNA fragments. Until now, three recombinants of PCV2 (P1, P2, and ZJ-R) have been identified during molecular epidemiological surveys using PCR [1,20,21]. Among these recombinants, we speculated that P1 is a recombinant virus formed between PCV2 and another virus (probably a retrovirus). This notion may prompt the reevaluation of PCV2 and PMWS and may also support the use of the same strategy to investigate the RNAs of PCV2 and PCV1 [22]. Its extremely small genome makes P1 a suitable tool for studying viral gene expression and replication. Despite its very small genome, P1 still displays a very complex transcription model, with eight viral RNAs identified in P1-transfected cells in this study. Two major RNAs (ORF1 and ORF3) were easily detected with northern blotting; some RNAs (ORF1, ORF2, ORF3, ORF4, ORF6, ORF7, and ORF8) were detected with PCR, and all eight RNAs (ORF1, ORF2, ORF3, ORF4, ORF5, ORF6, ORF7, and ORF8) were detected with RACE. Of the two P1-encoded transcripts detected with northern blotting, one (355 nt in size) is encoded on the viral (+) strand, and we believe that it is encoded by ORF3. The other abundant transcript (382 nt in length) is encoded on the viral (−) strand, and we assume that it is encoded by ORF1. We have shown that P1 mRNA is transcribed from both strands of the genomic molecule. ORF1, ORF2, ORF4, and ORF5 are transcribed in the leftward direction, and ORF3, ORF6, ORF7, and ORF8 are transcribed in the rightward direction.

The 5′ initiation site of ORF1 is located at nt 506 and the termination site is located at nt 125. The coding sequence starts at nt 493, terminates at nt 151, and encodes a protein of 113 amino acids. Comparison of the deduced N-terminal amino acid sequence of ORF1 with other proteins using the BLAST algorithm revealed extensive homology to the N-terminal domain of the PCV2 Cap protein, although the C-terminal amino acid sequence is low because there has been a frameshift in the ORF1.

ORF2 is initiated at nt 648 and terminates at nt 481. The coding sequence starts at nt 579, terminates at nt 492, and completely overlaps ORF3 transcribed in the opposite direction. The encoded protein has a sequence of 28 amino acids, with at least 86% sequence identity with the amino acid sequence of PCV2 ORF6.

ORF3 is initiated at nt 364 and terminates at nt 70. The coding sequence starts at nt 495 and terminates at nt 599. The protein of 34 amino acids has at least 91% sequence identity with the amino acid sequence of PCV2 ORF10.

The 5′ initiation site of ORF4 is located at nt 457 and the termination site is at nt 125. The coding sequence starts at nt 327, terminates at nt 279, and completely overlaps ORF1 in the same direction. A BLAST search of the GenBank database showed that it shares homology with a putative phosphatase from *Streptococcus mutans* [GenBank:WP_002277724].

ORF5 is initiated at nt 82 and terminates at nt 580. The coding sequence (16 amino acids) of ORF5 is between nt 52 and nt 1. A BLAST search of the GenBank database showed that it shares homology with an RNA-dependent RNA polymerase from a lymphocytic choriomeningitis virus [GenBank:AAX49344], an enzyme that catalyzes the replication of RNA from an RNA template.

The 3′ termination sites of ORF6, ORF7, or ORF8 are located at nt 458 and the 5′ initiation sites of ORF6, ORF7, and ORF8 are located at nt 232, 253, and 277, respectively. In ORF6, the coding sequence starts at nt 263 and terminates at nt 293. In ORF7, the coding sequence starts at nt 285 and terminates at nt 336. In ORF8, the coding sequence starts at nt 374 and terminates at nt 419. The ORF6, ORF7, and ORF8 proteins encode nine, 16, and 14 amino acids, respectively. A BLAST search of the GenBank database with the deduced ORF6, ORF7, and ORF8 amino acid sequences generated too many strong matches to easily determine their functions.

The large number of ORF1 transcripts suggests that it encodes a structural protein, e.g., a capsid protein. ORF1 also contains a conserved amino acid sequence at the N-terminus resembling that of a major structural protein of PCV2, so we investigated whether ORF1 of the P1 virus is a structural protein. In this study, a viral structural protein of 12.5 kDa was identified in the purified P1 particles, similar to the expressed ORF1 gene product. These results indicate that ORF1 encodes a 12.5 kDa protein that is the major structural protein of P1.

All the proteins encoded by the ORFs of the P1 virus are small proteins, except that encoded by ORF1. Even the products of ORF4, ORF5, ORF6, ORF7, and ORF8 can be considered peptides. These small proteins are known to participate in a wide variety of cellular processess [23]. The discovery and clarification of these small proteins will help to establish the unique roles they play in the host and the virus.

## Conclusions

Despite the small genome of the P1 virus, its transcriptional pattern is very complex. Although it is unclear whether most of these transcripts encode proteins, this study contributes to our knownledge of the biological properties of P1.
